# Prediction of Muscle Energy States at Low Metabolic Rates Requires Feedback Control of Mitochondrial Respiratory Chain Activity by Inorganic Phosphate

**DOI:** 10.1371/journal.pone.0034118

**Published:** 2012-03-28

**Authors:** Joep P. J. Schmitz, Jeroen A. L. Jeneson, Joep W. M. van Oorschot, Jeanine J. Prompers, Klaas Nicolay, Peter A. J. Hilbers, Natal A. W. van Riel

**Affiliations:** 1 BioModeling and Bioinformatics, Department of Biomedical Engineering, Eindhoven University of Technology, Eindhoven, The Netherlands; 2 Biomedical NMR, Department of Biomedical Engineering, Eindhoven University of Technology, Eindhoven, The Netherlands; 3 Netherlands Consortium for Systems Biology, Department of Biomedical Engineering, Eindhoven University of Technology, Eindhoven, The Netherlands; 4 Child Development and Exercise Center, Wilhelmina Kinderziekenhuis/University Medical Center Utrecht, Utrecht, The Netherlands; National Institute of Agronomic Research, France

## Abstract

The regulation of the 100-fold dynamic range of mitochondrial ATP synthesis flux in skeletal muscle was investigated. Hypotheses of key control mechanisms were included in a biophysical model of oxidative phosphorylation and tested against metabolite dynamics recorded by ^31^P nuclear magnetic resonance spectroscopy (^31^P MRS). Simulations of the initial model featuring only ADP and Pi feedback control of flux failed in reproducing the experimentally sampled relation between myoplasmic free energy of ATP hydrolysis (ΔG_p_ = ΔG_p_^o′^+RT ln ([ADP][Pi]/[ATP]) and the rate of mitochondrial ATP synthesis at low fluxes (<0.2 mM/s). Model analyses including Monte Carlo simulation approaches and metabolic control analysis (MCA) showed that this problem could not be amended by model re-parameterization, but instead required reformulation of ADP and Pi feedback control or introduction of additional control mechanisms (feed forward activation), specifically at respiratory Complex III. Both hypotheses were implemented and tested against time course data of phosphocreatine (PCr), Pi and ATP dynamics during post-exercise recovery and validation data obtained by ^31^P MRS of sedentary subjects and track athletes. The results rejected the hypothesis of regulation by feed forward activation. Instead, it was concluded that feedback control of respiratory chain complexes by inorganic phosphate is essential to explain the regulation of mitochondrial ATP synthesis flux in skeletal muscle throughout its full dynamic range.

## Introduction

The means by which oxidative ATP synthesis is controlled has remained an intensively studied topic during the past decades [1]. The first control scheme that was proposed involved a feedback signal of cellular ATP hydrolysis products, i.e. ADP and Pi [2]. More recently, a second control mechanism was proposed: i.e. parallel activation of cellular ATP demand and production (feed forward activation). It was hypothesized that parallel activation (feed forward regulation) of cellular ATP demand and production was essential to explain energy homeostasis [1], [3]. Since then, several sites of Ca^2+^ stimulation present in the mitochondrial network as well as a vast protein phosphorylation network controlled by Ca^2+^ signaling have been discovered [4]. These data provided further support of the parallel activation hypothesis However, although both control mechanisms have a firm basis in literature, it is still unclear to which extent each of these mechanisms contributes to the cellular energy homeostasis of the intact system (see e.g. [5], [6] vs. [1] and [7]). In addition, related questions, like e.g., the role of these control mechanisms in the development and progression of metabolic diseases, are considered important topics for future research [8].

Answering these questions requires a thorough understanding of the integrated system [9], [10]. Computational modeling has been proposed as an important research tool for keeping track of biological complexity and developing such ‘systems – level’ understanding [11], [12]. Although most models are constructed by integration of information obtained under *in vitro* experimental conditions, the goal of these models remains to represent *in vivo* conditions. It is therefore essential to test and improve them with *in vivo* data.

^31^P magnetic resonance spectroscopy (MRS) provides a non-invasive method for measuring metabolite dynamics (PCr, Pi, ATP) during rest, exercise and recovery conditions in human skeletal muscle [13]. Previously, ^31^P MRS was used to sample the transduction functions between regulatory metabolites (ADP, Pi) or thermodynamic potential (ΔG_p_ = ΔG_p_^o′^+RT ln [ADP][Pi]/[ATP]) and the oxidative ATP synthesis flux (J_P_) [14]. These transduction functions capture important characteristics of the regulation of oxidative phosphorylation *in vivo* and can therefore be applied for testing and validation of computational models of oxidative ATP metabolism.

The computational model of oxidative energy metabolism developed by Beard and coworkers [15] is among the most advanced models currently available. At first, it was developed to describe oxidative ATP metabolism in cardiac myocytes. At the moment, it has excellent performance in reproducing ^31^P MRS observed metabolite dynamics in cardiac cells [5], [6]. In addition, we showed that the model reproduced the transduction function between ADP and J_p_ recorded in skeletal muscle fairly well [14]. However, it has also been reported that at low respiration rates and corresponding ATPase fluxes (ATPase <0.2 mM/s) the model systematically underestimates ADP and Pi concentrations [14], [16], [17], which is most evident in predictions of the ΔG_p_ - J_p_ relation. These limitations are probably not a severe shortcoming for modeling of cardiac energetics. The normal physiological ATPase range of cardiac myocytes does not include these low fluxes. However, in case of skeletal muscle, or other excitable cell types, like neurons, the problem is considerably more significant, as these cells often experience low flux conditions.

It was studied if the observed model limitations are a result of inadequate parameterization; or alternatively, if the model is lacking essential control mechanisms. The latter will also have important physiological implications. Specifically, three hypotheses were tested: *i,* the model is not missing control mechanisms, but merely requires parameter optimization; *ii*, the missing control can be explained by addition of a substrate feedback mechanisms (by e.g. Pi) acting on a subset of model components; or *iii*, the missing control can be explained by addition of feed forward regulation (by e.g. Ca^2+^ signaling and protein phosphorylation) acting on a subset of model components.

To test these hypotheses, first, the relation between ΔG_p_ and J_p_ was obtained from high time resolution ^31^P MRS recordings of metabolite dynamics (PCr, Pi, ATP, ADP, pH) during recovery from exercise. The ΔG_p_ - J_p_ relation was favored for model testing above the PCr, Pi, ATP metabolite recovery dynamics for several reasons. PCr and Pi recovery dynamics are sensitive to cellular pH whereas ΔG_p_ - J_p_ is rather insensitive. Consequently, it is possible to analyze the ΔG_p_ - J_p_ relation without considering the effects of muscle acidosis. In addition, the transduction function provides a direct measure of the model error at a certain flux. Moreover, it was previously shown that failure of model predictions was observed best in predictions of ΔG_p_ [16], [17]. The relation was determined for healthy human subjects (control group) and two other populations, i.e., subjects with sedentary lifestyle and track athletes (validation datasets). Next, numerical analysis and model simulations were applied to test which of the three hypotheses could explain both control and validation datasets. The results rejected the solution of model re-parameterization or addition of regulation by a feed forward control mechanism. Instead, our findings provide new evidence in support of a substrate feedback related control mechanism that acts on the respiratory chain complexes, and at Complex III in particular, which regulates ΔG_p_ at low respiration rates in skeletal muscle.

## Results

### Model testing: analysis of model parameterization

**Figure 1:**
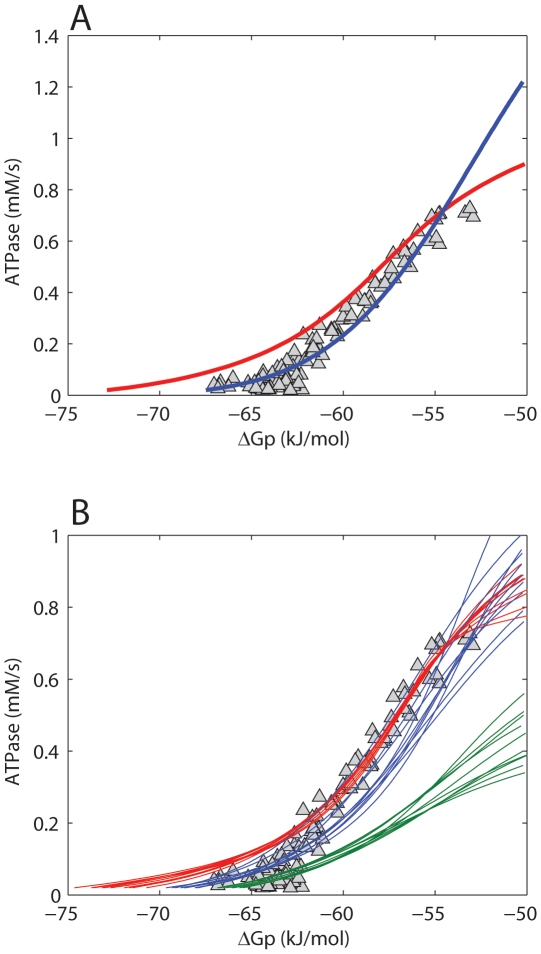
Testing model parameterization. Model predictions of the ΔG_p_ - J_p_ relation according to the initial model parameterization (red line) and after parameter optimization (blue line) (**A**). (**B**) shows the results of sampling of the model solution space by a Monte Carlo simulation approach. The 10 simulations with the best fit to all experimental data (blue lines), to high flux data points (ATPase >0.4 mM/s, red lines) and to low flux data points (ATPase <0.1 mM/s, green lines) are shown. Experimental data points are indicated by Δ. Data were taken from [14].

Model predictions of the ΔG_p_ - J_p_ relation were employed to test the hypothesis that the model harbors all essential control mechanisms, but requires parameter optimization. The experimentally sampled ΔG_p_ - J_p_ relation was obtained from a previous study [14], making use of ^31^P MRS during post exercise recovery in M. vastus lateralis of healthy human subjects. It was investigated which part of this dataset could be reproduced by the models' original parameterization. Predictions according to the model are shown in Figure 1A (red lines). While for ATPase >0.2 mM/s, model predictions and experimental data match well, for ATPase <0.2 mM/s the results show a clear discrepancy between predictions and data. These results confirmed that the original model fails to adequately describe rest and low exercise conditions.

Next, it was investigated if adjusting the model parameterization could improve model predictions. To this end, two different methods were applied. The first involved application of a parameter estimation algorithm (Levenberg – Marquardt minimization). In total 27 parameters were re-estimated. A list of these parameters and their original and re-estimated values is available in the supporting information (Table S1, columns: value original model; value optimized model). The mean squared error between model predictions and experimental data was used as objective function. For more details the reader is referred to the ‘Methods Section’. Figure 1A (blue line) shows model predictions vs. experimental data after optimization of parameter values. Although from a purely mathematical point of view these model predictions represent an improved fit of the model (the sum of squared errors decreased from 4396.5 kJ^2^/mol^2^ to 530.3 kJ^2^/mol^2^), additional simulations with the re-parameterized model predicted non – physiological behavior. Specifically, the predicted ΔG_p_ - J_p_ relation is expected to follow a sigmoidal relation in which the upper asymptote reflects maximal mitochondrial ATP synthetic flux (V_max_) [18], [19]. Model predictions show that the reparameterized model (Figure 1A, blue line) did not approach an asymptote in the high flux domain, but instead continued to rise. Consequently, the predicted V_max_ (∼2.5 mM/s) significantly overestimated previously reported values, which ranged between 0.62 and 0.83 mM/s [14]. Furthermore, the model failed in predictions of the experimentally observed ADP – J_p_ transduction function (simulation results provided in supporting information, Figure S2). For example, the predicted [ADP] at half maximal flux (K_50ADP_), was ∼0.2 mM, which is about tenfold larger than calculated from the experimentally sampled ADP – J_p_ relation [14]. It was also tested if including both the ΔG_p_ - J_p_ and ADP – J_p_ data in the parameter optimization procedure could improve the performance of the model. However, in this case, the transduction function of the reparameterized model became very similar to the initial model and was rejected because it failed to describe rest and low exercise conditions (simulation results provided in supporting information, Figure S3). In the previous approach large changes of model parameter values were allowed, resulting in up to 20 fold changes of estimated parameter values (see supporting information Table 1). Many of the optimized parameters were originally derived from other computational studies and consequently have no firm experimental basis. Therefore, at itself, the required changes in parameter values provide no basis to reject the possible solution of model reparameterization. However as a result of these large changes in parameter values non – physiological model behavior was observed, which, did provide evidence to reject the model. In an attempt to study model predictions which were more constrained to the physiological domain, model behavior was sampled for a large number of parameter sets around the initial parameterization. In this approach the parameter values were more strongly constrained with the aim of keeping the model in the physiological relevant domain. Next, a Monte Carlo simulation approach (10,000 simulations) was applied to randomly select parameter values within the range of 0.1–2 times the values of the initial model parameterization (uniform distribution). Figure 1B, shows the 10 simulations with the best fit to all experimental data (blue lines), to the high flux data points (ATPase >0.4 mM/s, red lines) and to the low flux data points (ATPase <0.1 mM/s, green lines). These results indicated that the model can either fit the high flux domain experimental data (red lines) or the low flux domain experimental data (green lines), but cannot reproduce all data points with a single set of parameters (blue lines).

**Table 1:** Overview of mitochondrial model parameters included in the metabolic control analysis.

Parameter name	Description
*X_DH_*	Dehydrogenase activity
*X_C1_*	Complex I activity
*X_C3_*	Complex III activity
*X_C4_*	Complex IV activity
*X_F1_*	F_o_F_1_-ATPase activity
*X_ANT_*	ANT activity
*X_PiHt_*	H^+^/Pi^−^ cotransporter activity
*X_Hle_*	H^+^ leak activity
*Mito_Adn_*	Mito. outer membrane permeability to nucleotides
*Mito_Pi_*	Mito. outer membrane permeability to Pi
*X_AtC_*	Cytoplasm ATPase activity

On the basis of the results above it was concluded that reparameterization of the model did not suffice to reproduce the experimental data. An alternative explanation would be that the model was lacking essential regulatory control mechanisms. This possibility was investigated in the remainder of the study.

### Mathematical analysis of model control points: metabolic control analysis

A first essential step in testing hypotheses of additional control was to identify the subset of model components that were most likely involved. Metabolic control analysis (MCA) was applied to identify components influencing ΔG_p_ at low flux conditions. MCA has been described in multiple review papers (see e.g. [20]). In brief, MCA is a quantitative framework for relating steady state fluxes or concentrations in a biochemical network to properties (control coefficients) of the networks individual components. The control coefficients reflect the sensitivity of model predictions (steady state flux or concentrations) to a change in the activity (V_max_) of an individual network component. The model parameters that were included in the MCA are listed in Table 1. The list was constructed by selecting all model parameters representing enzyme activities (V_max_). Enzyme activity parameters of creatine kinase, adenylate kinase, the mitochondrial K^+^/H^+^ exchanger and magnesium binding fluxes were excluded from the analysis since at steady state the fluxes through these enzymes were zero and consequently, control coefficients could not be calculated. The control coefficients were calculated for resting skeletal muscle (ATP demand: 0.01 mM/s [21]) because for this condition model predictions failed most dramatically (Figure 1). In this section only the results relevant for the present investigation are described; the entire matrix of flux and concentration control coefficients is available in the supporting information (Tables S2 and S3).

**Figure 2:**
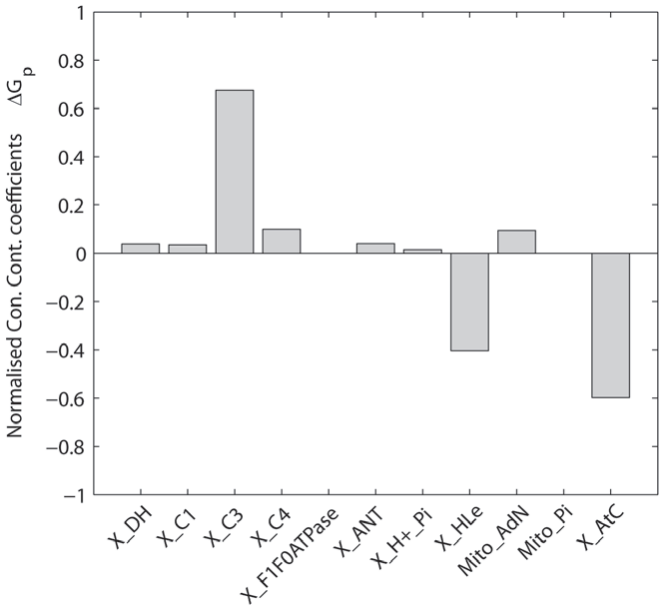
Normalized concentration control coefficients for cellular phosphate potential (ΔG_p_). Concentration control coefficients were calculated for cellular ATPase rate 0.01 mM/s.

According to the theory of metabolic control analysis, the concentration control coefficients sum to 0 [20]. Normalized concentration control coefficients were calculated for ΔG_p_, Figure 2. The concentration control coefficients were normalized by scaling the sum of positive control coefficients to 1. Consequently, the sum of negative concentration control coefficients summed to −1. The positive concentration control of ΔG_p_ was for 60% located at complex III, whereas the remaining part of the positive control was distributed among the other components of the network. The negative control was shared between proton leak (control coefficient −0.35) and cellular ATP demand (control coefficient −0.65). Complex III, the proton leak and cellular ATP demand were therefore proposed as possible candidates for additional regulation. Additional simulations revealed that indeed by adjusting the proton leak flux it was possible to predict the experimentally observed ΔG_p_ at rest. However, it required increasing the proton leak flux until over 99 percent of all protons entered the matrix through the leak. The physiological range of the proton leak in skeletal muscle is between 35 and 50% [22]. Since the proton leak at rest accounted already for 50% of the proton flux into the mitochondrial matrix, this network component was ruled out as a potential site of additional regulation. Simulations also confirmed that by increasing cellular ATP demand it was possible to reproduce the experimentally observed ΔG_p_ at rest (−64 kJ/mol). However, this could only be achieved when the cellular ATP demand at rest was increased 15-fold, from 0.01 mM/s to 0.15 mM/s. The cellular ATP demand flux in human skeletal muscle at rest has however been measured accurately by a variety of experimental methods [23]. The reported values range from 0.002 to 0.02 mM/s, median value: 0.01 mM/s. Increasing basal ATP demand to 0.15 mM/s corresponded to non-physiological conditions. It was therefore chosen to set the basal ATP consumption flux to the experimentally observed value (0.01 mM/s). Consequently, in addition to the proton leak flux, also basal ATP demand flux was ruled out as possible solution for improving model predictions. It was therefore chosen to focus on complex III as the primary site of additional control.

### Hypotheses of additional regulation

The original model provided insufficient control to capture the regulation present in skeletal muscle *in vivo*. In this section two alternative model configurations are defined, according to the two currently leading hypotheses: i.e. 1.) substrate feedback regulation [6], and 2.) feed forward activation (parallel activation) mediated by protein phosphorylation [1]. For both configurations, complex III was selected as the site of the additional control.

#### Substrate feedback regulation

The original model already included substrate feedback regulation of the dehydrogenases and complex III, which was described by a phenomenological hyperbolic activation term as a function of matrix Pi concentration. In the new model this term was substituted by a Hill equation which can describe both a hyperbolic relation (*nH = 1*) as well as a sigmoidal relation (*nH>1*). The Hill equation captured a wider range of regulatory functions and thus a wider range of potential control mechanisms. The new flux equation for complex III was defined as follows:
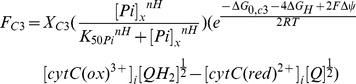
(1)


#### Feed forward activation (parallel activation)

In the feed forward activation model two states of complex III were defined; a phosphorylated active state (*X_phos_*) and dephosphorylated inactive state (*X_dephos_*). The flux through these model components (complex III, *Eqn 2*) was calculated as the weighted sum of the fluxes through the phosphorylated active (weighting parameter *F_A_*) and the dephosphorylated inactive states (weighting parameter *F_IA_*) of complex III. k*ineticEq_C3_* denotes the kinetic description of the flux through complex III according to the original model.
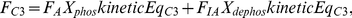
(2)where
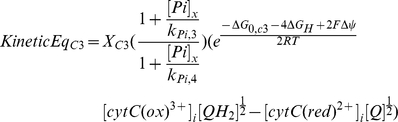



The activity of the regulatory kinase-phosphatase system controlling the fraction of complex III in the phosphorylated and the dephosphorylated state was modeled with parameters *K_on_* and *K_off_* yielding differential equations *Eqn 3* and *Eqn* 4. According to the parallel activation hypothesis, the equilibrium between the phosphorylated and dephosphorylated states (*K_on_/K_off_*) was modeled as a function of cell energy demand. Hereto *K_off_* was described by *Eqn 5*, in which the energy demand of the cell was expressed by the cytoplasmic ATPase flux *J*_AtC_

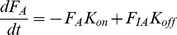
(3)

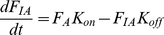
(4)

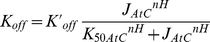
(5)Both model configurations are summarized in Table 2. Newly introduced model parameters were estimated based upon the experimental data of the ΔG_p_ - J_p_ transduction function recorded in healthy, normally active human subjects (Table 3 and 4). Full details of the parameterization procedure are provided in the ‘Methods’ section.

**Table 2:** Summary of model configurations.

Model configuration	Regulatory site	Regulatory mechanism
*i*	Complex III	Substrate feedback
*ii*	Complex III	Parallel activation

**Table 3:** Parameter values model configuration *i*, substrate feedback at complex III.

Parameter name	Value (mean+/−SD)	Unit
*X_CIII_*	54.24+/−4.28	mol s^−1^ M^−3/2^ (L mito)^−1^
*nH*	4.02+/−0.35	unitless
*K_50Pi_*	6.43+/−0.52	mM

**Table 4:** Parameter values model configuration *ii*, parallel activation at complex III.

Parameter name	Value (mean+/−SD)	Unit
*X_A_*	1.46+/−0.21	unitless
*X_IA_*	0.030+/−0.010	unitless
*nH*	1.35+/−0.11	unitless
*K_50AtC_*	0.094+/−0.015	mmol (L cell water)^−1^ s^−1^
*K_on_*	0.033+/−0.006	s^−1^
*K^′^_off_*	52.64+/−17.4	s^−1^

**Figure 3:**
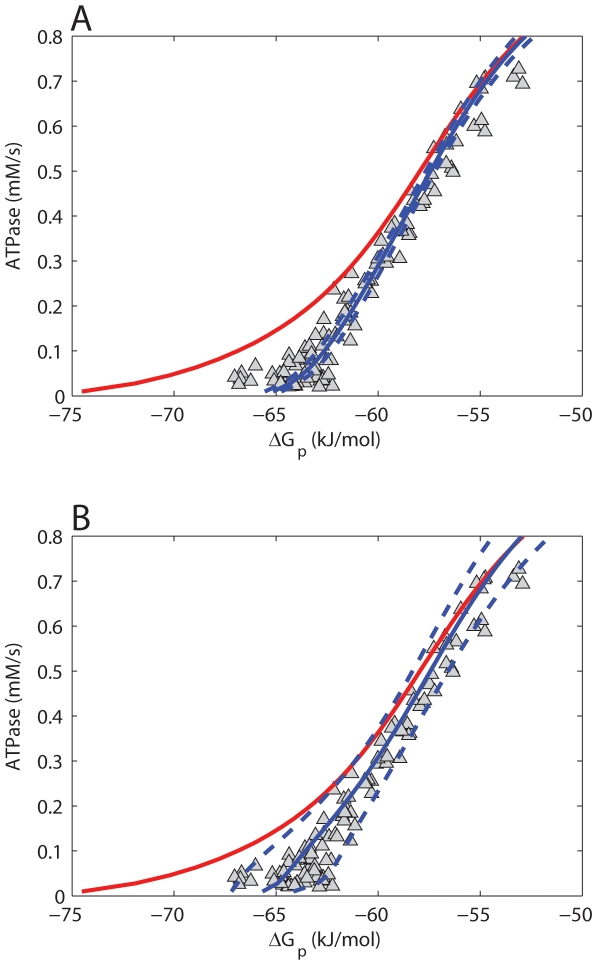
Model predictions of the ΔG_p_ - J_p_ relation. Model predictions of the relation between cellular phosphate potential (ΔG_p_) and mitochondrial ATP synthetic flux (ATPase) according to model configuration *i* (metabolic feedback regulation) (**A**), model configuration *ii* (regulation by parallel activation) (**B**). Model predictions are indicated as the mean (solid blue line) and STD (dotted blue line) of the 1000 simulations that were run in a Monte Carlo approach. Predictions of the original model are shown in red. Experimental data points are indicated by Δ. Data points were obtained from [14].

It was tested if the two model configurations could reproduce the ΔG_p_ - J_p_ relation after parameter optimization. These simulations were run in a Monte Carlo simulation approach to probe the effect of the uncertainty in the newly introduced model parameter values on model predictions. 1000 simulations were run and parameter values were randomly selected from the 95.4% confidence interval (mean+/−2*SD) of normal distributions with mean and SD as reported in Tables 3 and 4. The selected parameters were limited to +/−2×SD to ensure no negative parameter values were drawn. The calculations were performed for both model configurations and results are shown in Figure 3A,B. The solution space of the model was represented by the mean (solid blue line) and standard deviation (dashed blue line) of the 1000 simulations obtained in the Monte Carlo approach. In each sub-figure the simulation of the original model lacking additional regulation is indicated in red. The solution space of the substrate feedback model (configuration *i*) was more constraint compared to the parallel activation model (configuration *ii*), which is probably a result of the smaller number of parameters included in the substrate feedback model configuration. Nevertheless, the mean of the solution space was for both model configurations consistent with the experimentally observed ΔG_p_ - J_p_ relation.

### Mitochondrial redox state, inner membrane potential and mitochondrial ADP sensitivity

**Figure 4:**
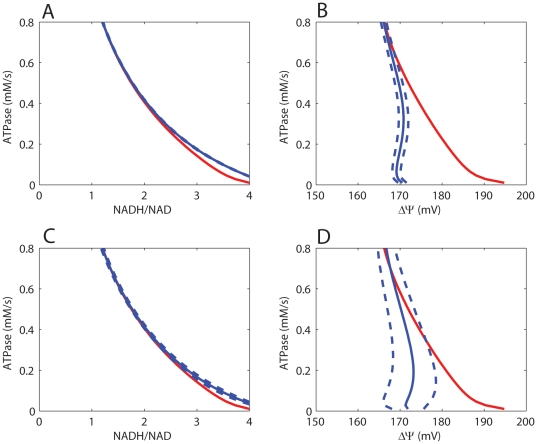
Model predictions of mitochondrial redox state (NADH/NAD) and membrane potential (ΔΨ). Model predictions of mitochondrial redox state (NADH/NAD) and membrane potential (ΔΨ) at different cellular ATP demand fluxes according to model configuration *i* (**A–B**) and model configuration *ii* (**C–D**). Model predictions are indicated as the mean (solid blue line) and SD (dotted blue line) of the 1000 simulations that were run in a Monte Carlo approach. Predictions of the original model are shown in red.

Because both model configurations could reproduce the control dataset, it was next investigated if model predictions of important mitochondrial state variables were within physiological range. Figures 4A–D show model predictions of the mitochondrial redox state (J_p_ - NADH/NAD) and membrane potential (J_p_ - ΔΨ) at different cellular ATP demand fluxes. These simulations were also run in a Monte Carlo approach to probe the effect of uncertainty in model parameterization (SD reported in Table 3, 4). Results of the original model lacking any additional regulation are indicated in red (Figure 4). Compared to the original model, the newly added regulatory elements decreased the inner membrane potential (ΔΨ) at low flux conditions. For both model configurations predictions of ΔΨ and NADH/NAD remained within their physiological range (150–200 mV, 0.3–100 respectively). Model predictions of J_p_ - NADH/NAD were very similar for both model configurations (blue) as well as the original model (red). This result is explained by the calculated concentration control coefficients. The positive control is dominated by the dehydrogenase flux (X_DH_). The negative control is primarily provided by cellular ATP demand and proton leak flux. The flux equations of these model components are left unchanged compared to the original model. As a result, only minor differences in predictions of the J_p_ - NADH/NAD relation were observed. In addition, model predictions of J_p_ - NADH/NAD relation are in good correspondence with experimental observations in isolated mitochondria [24]. The experimental data indicate an NADH/NAD ratio at State 3 respiration of about 1. NADH/NAD ratio was found to increase at lower ATP turnover fluxes eventually approaching a value of 100 at State 4 respiration. In addition, it was tested if predictions according to the two model configurations reproduced a sigmoidal ΔG_p_ - J_p_ relation, physiological V_max_ value and experimentally observed ADP - J_p_ relation. It was concluded that both model configurations successfully reproduced these physiological characteristics and data (results provided in supplementary materials, Figure 2).

### Model testing against independent data

**Figure 5:**
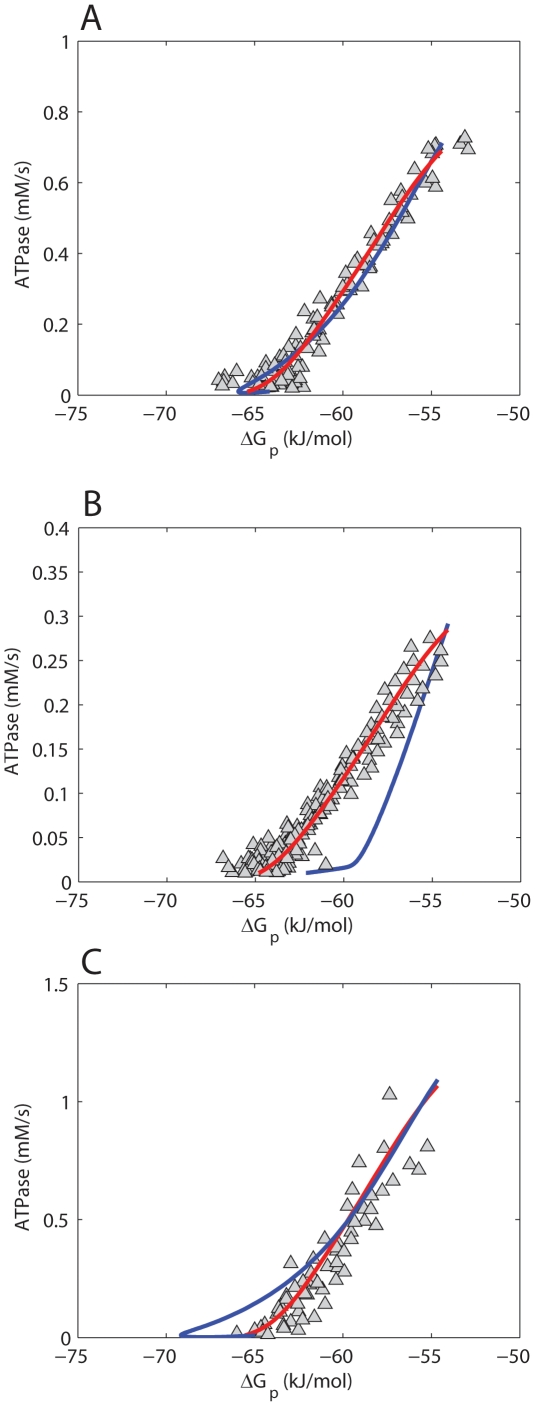
Model predictions for phenotypes with different mitochondrial densities. Model predictions of the ΔG_p_ - J_p_ relation during post exercise recovery. Results are shown for normally active healthy subjects (**A**), sedentary subjects (**B**), athletes (**C**). Experimental data are indicated by Δ. Model predictions according to the substrate feedback model (model configuration *i*) are indicated by a red line. Model predictions according to the parallel activation model (model configuration *ii*) are indicated by a blue line.

Based on predictions of physiological end-points (NADH/NAD, ΔΨ) it was not possible to discriminate between the model configurations. Furthermore, steady state model predictions of both configurations were consistent with the experimentally observed ΔG_p_ - J_p_ relation (Figure 3). The experimental data was however recorded during post exercise recovery period. It was therefore also tested if model predictions derived from simulations of post exercise recovery were consistent with the data. The data of normally active subjects was already used for parameter estimation and cannot be considered an independent dataset. Therefore, in addition, also independent data of athletes and obese sedentary subjects was used. A well known difference in phenotype between these groups is the mitochondrial density in skeletal muscle; the athletes having more mitochondria, the sedentary subjects having less. Model predictions according to both configurations are dependent on settings for the mitochondrial volume density. It was tested if the model could reproduce the data for all these phenotypes by adjusting only the mitochondrial volume density. All other parameters, included the ones of the newly introduced regulatory mechanisms were left unchanged. The results of these simulations are shown in Figure 5A–C. The substrate feedback model (red lines) could reproduce the experimental data for all three phenotypes. In contrast, the parallel activation model (blue lines) could only reproduce the experimental data of the healthy, normally active human subjects and failed in reproducing any of the two independent datasets.

In addition, it was also investigated if the parallel activation model could reproduce the two independent datasets by adjusting its parameterization. In fact, this was indeed possible, but only in the specific cases that parameters *K_on_* and *K_off_* were tuned to make the time constant of deactivation of the mitochondria (transition of phosphorylated, activated complex III into the non-phosphorylated inactive state, *A→IA*) similar to the time constant of metabolic recovery (PCr, Pi recovery). The experimentally observed time constant of metabolic recovery was quantified by a mono-exponential fit to the PCr recovery data. The corresponding time constant was 82+/−6.4 s, 15+/−6.5 s and 28+/−5.4 s for the sedentary, athletes and healthy control subjects, respectively (mean+/−SD). The predicted time constant of mitochondrial deactivation (*A→IA*) was 29.5 s (mono-exponential fit), which matches the experimentally observed time constant of metabolic recovery of the healthy control subjects well (28+/−5.4 s). After adjusting the values *K_on_* and *K_off_* to fit the data of sedentary subjects or athletes, the predicted time constant of deactivation was 91.6 s and 14.7 s, respectively. These values are again similar to the time constant of metabolic recovery measured for these subjects (82+/−6.4 s and 15+/−6.5 s, respectively). It was concluded that the model could only reproduce the ΔG_p_ - J_p_ relation of a dataset if the time constant of metabolic recovery matched the time constant of deactivation of the mitochondria. However, in these cases, the model failed in reproducing the other two datasets. The substrate feedback model could predict all datasets without re-parameterization of the regulatory mechanism. The substrate feedback model was therefore defined as the most robust and selected as best model configuration and thus the most likely hypothesis.

### Model testing against ^31^P MRS observed metabolite recovery dynamics

**Figure 6:**
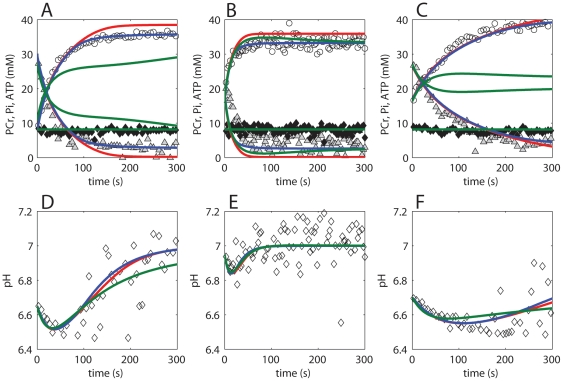
Model predictions versus ^31^P MRS observed metabolite dynamics during post exercise recovery period. Experimental data and model simulations of a healthy control subject (**A,D**), athlete (**B,E**) and subject with a sedentary lifestyle (**C,F**) are shown. Model simulations according to the original model, model configuration *i* and model configuration *ii* are indicated by red, blue and green lines respectively. Experimental data of PCr, Pi, ATP and pH are indicated by open circles, grey triangles, closed diamonds and open diamonds respectively.

Model simulations were tested against ^31^P MRS observed recovery dynamics (PCr, Pi, ATP, pH). For each population (control subjects, atheletes, subjects with sedentary lifestyle) an individual dataset was selected. It was necessary to test model simulations against an individual data instead of the mean+/−SD of all recorded data, because these datasets were characterized by differences in end-exercise pH and level of PCr depletion. In Figure 6, model simulations for the original model (red lines), model configuration *i* (blue lines) and model configuration *ii* (green lines) are compared to PCr (open circles), Pi (grey triangles), ATP (closed diamonds), and pH (open diamonds) recovery dynamics. Full details of the simulation protocol are provided in the ‘Methods’ section. Model predictions according to the original model failed in predicting the correct PCr and Pi levels at fully recovered state for the control subject and the athlete dataset. The parallel activation model (model configuration *ii*) could not reproduce metabolite dynamics for the control subject and the subject with sedentary lifestyle. Remarkably, model configuration *ii* reproduced the ΔG_p_ - J_p_ relation of the control data well (Figure 5), but it failed in reproducing the metabolite (PCr, Pi) dynamics of the same dataset (Figure 6). This result is explained by the effect of end - exercise pH. The calculations for the ΔG_p_ - J_p_ relation were performed for resting pH (7.05) because the relation was insensitive to changes in pH. The calculations of the metabolite dynamics (PCr and Pi recovery) are however sensitive for end – exercise pH, which was therefore taken into account in the simulations. The substrate feedback model (model configuration *i*) could reproduce the dynamics of all datasets. The results show this model configuration can also reproduce the time constant of metabolic (PCr, Pi) recovery for the different datasets. These simulations therefore provide again further evidence that the substrate feedback model is the best model configuration.

## Discussion

The main result of this investigation was twofold. First, it was identified that the control mechanisms captured by a detailed biophysical model of oxidative phoshporylation in human skeletal muscle could not explain the empirically observed ΔG_p_ - J_p_ relation, even not after parameter optimization. Second, the results support the hypothesis that substrate feedback control of the respiratory protein complexes plays an important role in the regulation of mitochondrial ATP synthesis in skeletal muscle by controlling ΔG_p_ at low ATPase rates in muscle. These main results as well as several methodological considerations are discussed.

### Methodological considerations

The computational model that was used as a basis for this investigation builds upon on a series of previously published models [14], [15], [17]. A possible concern for applying this model could be the use of a homogenous unit to represent skeletal muscle tissue. It is well known that skeletal muscle is a heterogeneous tissue in which distinct cell types are present [25]. These cell types differ in size, force generating capacity, but also mitochondrial density. In the current model this was not taken into account; skeletal muscle tissue was modeled by a single cell in which an averaged mitochondrial density was represented. It was therefore investigated in more detail if this choice affected the study outcome. Three different cells were defined, each with a different mitochondrial density. The mean density was equal to the value used in the single cell model. Predictions of the ΔG_p_ – J_p_ relation were identical when comparing the average of the three cells and the single cell model. From these results it was concluded that this specific model assumption did not affect the overall outcome of this study.

Another important model assumption was that ATP was produced purely through oxidative processes. This choice was justified by limiting the analyses to data recorded during post exercise recovery period. For this period it is well established that the PCr dynamics reflect almost purely oxidative ATP synthesis [21], [26]–[28].

The current analysis was conducted under the assumption that changes in cellular pH as a result of e.g. lactate acidosis did not significantly affect the ΔG_p_ – J_p_ relation. This assumption was verified by analysis of the experimental data. The data of healthy normally active subjects was obtained during multiple exercise bouts of varying length and intensity. As a result, the ΔG_p_ – J_p_ relation was sampled for varying conditions of end – exercise pH. It was investigated if varying conditions of cellular pH influenced the observed ΔG_p_ – J_p_ relation (results provided in supporting information, Figure S1). The results indicated that the ΔG_p_ – J_p_ relation was insensitive to cellular pH, which validated the model assumption.

The model of oxidative phosphorylation that was chosen as a basis for the analysis was previously derived from a model parameterized based upon data of cardiac mitochondria [15]. The conversion to a skeletal muscle model mainly comprised adjusting metabolite pool sizes and structural parameters (e.g., mitochondrial volume percentage). In contrast, the kinetic parameters of oxidative phosphorylation remained unchanged. An assumption underlying these studies therefore is that the parameterization derived from the cardiac mitochondria is also representative for skeletal muscle. The experimental data used to parameterize the original model were taken from Bose et al. [29]. Bose et al. also reported that they conducted the experiments with mitochondria collected from skeletal muscle. It was reported that the results of the skeletal muscle mitochondria were very similar to that of cardiac mitochondria. In addition, the assumption is further supported by proteomics studies, which show that cardiac and skeletal muscle mitochondria are very similar [30]. Furthermore, it was tested if failure of model predictions at low ATP turnover rates could be a result of one or more of the parameter values to be incorrect for skeletal muscle. Optimizing model parameters could however not solve the problem. Next, the results of the metabolic control analysis were used to investigate which model parameters influenced predictions of ΔG_p_. These results showed that the majority of the concentration control was located at complex III. Conversely, they indicated that model predictions were insensitive to changes in other parameters. It was therefore concluded that possible small deviations in parameter values as a result of the cardiac origin of the model did not affect the overall outcome of the study. Nevertheless, these results do not rule out the possibility that in future studies other model predictions actually are sensitive to multiple (other) kinetic parameters. Analysis of the differences in behavior of skeletal muscle and cardiac mitochondria and translation of these differences to models' parameterization may therefore become an important topic of future research.

Some physiological parameters that were not modeled in detail were taken into account in the analysis implicitly. For example, nutrient supply was captured by the lumped dehydrogenase flux (X_DH_) and oxygen availability will affect mitochondrial dynamics through complex IV flux (X_C4_). The control coefficients determined for X_DH_ and X_C4_ therefore also implicitly reflect control of these physiological parameters.

The two models used to describe the additional regulation are of phenomenological nature. For testing and evaluating of these concepts the models were found very insightful. However, one should remain careful with the deduction of statements related to the molecular mechanisms of the regulation. For example, although the results clearly indicate the signal modulating respiratory chain activity to be related to cellular substrate levels, it cannot be ruled out that molecular implementation of this regulation involves e.g. protein (de)phosphorylations or other post translational modifications. The results of this study therefore do not contradict with recent evidence of the vast mitochondrial protein phosphorylation network [1].

### Regulation of oxidative ATP production in skeletal muscle

It was concluded that a substrate feedback related control signal provided the best explanation for the additional regulation. The evidence supporting this conclusion was twofold: the model configuration representing the substrate feedback control mechanism could reproduce both the control dataset (healthy volunteers) and the validation datasets (subjects with sedentary lifestyle and track athletes) without re-parameterization, whereas the parallel activation model could not. Secondly, the parallel activation model could reproduce the data of humans with a sedentary lifestyle or track athletes if re-parameterized. However, this occurred only in the specific case that parameter settings caused the time constant of deactivation (transition of phosphorylated, activated complex III into the non-phosphorylated inactive state) to match the time constant of metabolic recovery. This result indicated that the regulatory mechanisms could still involve post translational modifications (e.g. protein phosphorylation) but that the key control signal is probably closely linked to substrate levels (e.g. Pi, ADP, ADP/ATP). In a previous study we proposed that the order of the ADP sensitivity provides a tractable validation criterion for evaluation of computational models of oxidative ATP metabolism [14]. Experimentally a second order Hill - coefficient was observed (1.9+/−0.2) [14]. Analysis of the computational model revealed that the mitochondrial ultra-sensitivity to ADP was primarily controlled by the kinetic parameters of the adenine nucleotide transporter (ANT). Reformulation of the ANT kinetics increased the Hill - coefficient of the model from 1 to 1.5. Although this was an important step forward, the computational model still underestimated the experimentally observed Hill - coefficient (1.5 vs. 1.9, respectively). It was concluded that the kinetic mechanisms required for the remainder of the difference in Hill - coefficient were not yet included in the model and remained to be identified. Specifically, multisite Pi activation of the mitochondrial network was ruled out as mechanism because it was already explicitly incorporated in the model. However, in the new model, the Pi activation term of the Complex III flux description was updated. The modification of the Pi activation term could also affect the models corresponding Hill – coefficient. In fact, model simulations revealed that the changes applied in the new model increased the Hill - coefficient to 1.8, which is close to the experimental observations (1.9+/−0.2). On this basis, it was concluded that in addition to the kinetic parameters of ANT, Pi modulation of respiratory chain activity contributes to the model predictions of second order kinetics of mitochondrial sensitivity to ADP.

The regulation of skeletal muscle oxidative ATP metabolism has also been investigated extensively by Korzeniewski and colleagues (see e.g. [31]–[33]). The model that was developed and updated in these studies overlaps in part with the model used in the current investigation: i.e., part of the models topology and some flux equations and parameter values are identical. Interestingly, the studies by Korzeniewski and colleagues point towards a principal role for parallel activation in the regulation of oxidative phosphorylation in skeletal muscle [33]. Although, at first, this result seems conflicting, Korzeniewski and colleagues evaluated many other quantitative but also qualitative characteristics of skeletal muscle energy metabolism (like e.g., PCr overshoot behavior or asymmetry between PCr on - off kinetics). In the present study we focused on ^31^P MRS observed metabolite dynamics, and, in particular the ΔG_p_ – J_p_ relation. Our results however do not rule out the possibility that regulation by parallel activation is essential to explain other characteristics of energy metabolism in muscle. At the moment, these models share the same ANT flux equation. Previously, we concluded that this particular component has a dominant role in controlling the model sensitivity to ADP [14]. Moreover, it was concluded that the K_50ADP_ (i.e., [ADP] at half maximal velocity) derived from predictions of the ADP – J_p_ relation should be increased tenfold by adjusting ANT parameter *θ* to match the experimentally observed ADP – J_p_ relation. Adjusting the ADP sensing of the model also amplified the models' sensitivity to control by substrate feedback regulation (ADP, Pi). This step in model development provides an explanation for why the current model did not require multi step parallel activation for reproducing the experimentally observed ΔG_p_ – J_p_ relation.

The physiological implications of the added regulation on e.g. membrane potential and mitochondrial redox state were also explored. The model predicted a decreased inner membrane potential at low flux conditions. For instance, under resting conditions, the membrane potential dropped by 25 mV as a result of the added regulation. These predictions may provide a clue about the functionality of the regulation. A high mitochondrial membrane potential is believed to be a major source of cellular ROS production [34] and corresponding cellular damage. There is an increasing amount of evidence that mitochondria regulate membrane potential by e.g. the concentration of uncoupling proteins in the inner membrane [35], [36]. We speculate that the proposed Pi regulation of respiratory chain activity has a similar function: it prevents high membrane potentials under low flux conditions protecting the cells against excessive ROS production.

### Significance and future prospective

It is concluded that explaining the experimentally observed relation between ΔG_p_ and J_p_ in skeletal muscle *in vivo* requires increasing the control of the Pi activation term of complex III in a detailed model of oxidative ATP metabolism. The significance of this result is twofold. First, it provides new evidence supporting a dominant role for substrate feedback regulation in the control of mitochondrial ATP synthesis in skeletal muscle. Second, the proposed adaptations provide an important step towards developing a computational model of ATP metabolism in skeletal muscle representing *in vivo* conditions. The updated model was shown to reproduce ^31^P MRS observed metabolite dynamics throughout the entire dynamical range of ATPase fluxes in skeletal muscle *in vivo*. To the best of our knowledge, this is the first detailed computational model that is quantitatively consistent with ^31^P observed metabolite dynamics throughout this full range. Hence, it provides an improved basis for future studies of energy metabolism in muscle. From this viewpoint, application of the model may not remain limited to healthy subjects, but may also include analysis of for example mitochondrial (dys)function related to obesity, type 2 diabetes or aging. Specifically, model simulations can quantify the contribution of changes in specific physiological parameters (e.g. mitochondrial content, enzyme activities) observed in these patients to ^31^P MRS recordings of mitochondrial function *in vivo* (time constant of post exercise PCr recovery). This study can test the relevance of the current model for the analysis of metabolic diseases in humans.

## Methods

### Ethics statement

The experimental data recorded in healthy human control subjects and subjects with sedentary lifestyle were obtained from previous investigations [14], [37]. The data recorded in athletes has not been published before. For all experiments, the nature and risks of the experimental procedures were explained to the subject. All gave their written informed consent to participate to the study, which conformed the standards set by the Declaration of Helsinki and was approved by the local Medical Ethics Committee of the Máxima Medical Centre, Veldhoven, The Netherlands.

### Experimental data

#### Healthy, normally active control subjects

The experimentally sampled ΔG_p_ – J_p_, relation was obtained from human quadriceps muscle of healthy, normally active subjects using *in vivo* ^31^P NMR spectroscopy. Full details regarding the methods of these experiments are described elsewhere [14]. In brief, subjects (n = 6) performed single-leg knee extension every 1.5 s in supine position. The experimental protocol consisted out of 2 min rest followed by incremental ramp exercise protocol of different duration, followed by a recovery period. ^31^P MRS recordings of PCr, Pi ATP and pH levels were conducted at 6 seconds time resolution (repetition time: 3 s, no. of averages: 2, spectral width: 2000 Hz, no. of data points in the FID: 1024). Spectra were fitted in time domain by using a non-linear least square algorithm in the jMRUI software package. Absolute concentrations of the metabolites were determined after correction for partial saturation and using the ATP concentration as internal standard (assuming that [ATP] is 8.2 mM at rest [38], [39]). Intracellular pH was calculated from the chemical shift difference between the Pi and PCr resonance [40]. The free cytosolic ADP concentration was calculated from pH and [PCr] using creatine kinase equilibrium constant (K_eq_) of 1.66×10^9^ M^−1^ [41] and assuming that 15% of the total creatine is unphosphorylated at rest [42], using Eqn. 6:
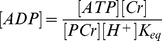
(6)


The molar free energy of cytosolic ATP hydrolysis was calculated according to Eqn. 7, where 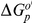
is −31.8 kJ/mol at 37°C. [ADP] and [ATP] represent the magnesium-free concentrations of these metabolites. 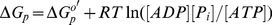
(7)The PCr recovery time course was fitted to a mono-exponential function. The PCr resynthesis rate was calculated from the derivative of the fitted PCr recovery time course. During recovery from exercise PCr is resynthesized purely oxidatively [21], [26]–[28]. Because the creatine kinase reaction is much faster than oxidative ATP production [43] the derivative of the fitted PCr recovery time course reflects mitochondrial oxidative phosphorylation flux (J_p_). The experimental data (ΔG_p_ – J_p_) used for testing of the computational model were obtained from the post exercise recovery dynamics.

#### Subjects with sedentary lifestyle

The data of sedentary humans used for model validation were obtained from de Feyter et al. [37]. This dataset was recorded using the same methodology as described above The inter – subject difference in mitochondrial capacity in the study by de Feyter et al. was rather large (range: 0.31–0.82 mM/s). It was chosen to select the data of 2 subjects with the lowest mitochondrial capacity. As a result the scatter of data points decreased, making the dataset more appropriate for rigorous model testing, while the ΔG_p_ – J_p_, relation was still sufficiently sampled (>300 samples). Analysis of muscle biopsy samples taken from the same subjects [44] showed that the decreased mitochondrial capacity correlated well with *in vitro* measures of mitochondrial content (CS and SDH activity). These results provided additional verification that the selected sedentary subjects had a decreased mitochondrial content.

#### Athletes

The data recorded in athletes has not been published before. The applied methodology was identical to the two other datasets (normally active, sedentary) [14], with the only exception being that for this study a bicycle ergometer was used and the sample time of the measurements was decreased from 6 to 3 s. Details of the ergometer can be found elsewhere [45]. The track athletes (age: 22+/−2 mean+/−SD, n = 3) participated in national and international level competition and trained for more than 8 hours a week.

### Model description

**Figure 7:**
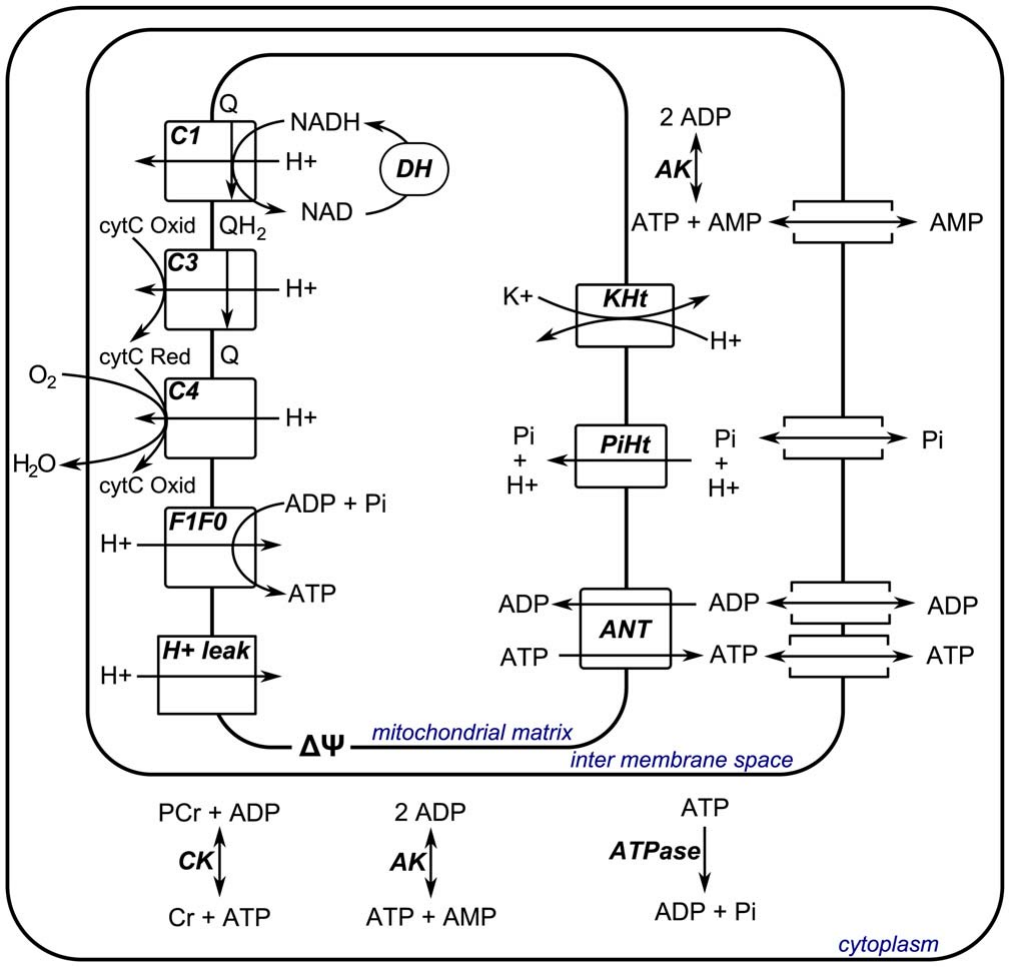
Schematic representation of the computational model of skeletal muscle energetics. Abbreviations denote: dehydrogenases (DH), complex I (C1), complex III (C3), complex IV (C4), F_1_F_0_ATPase (F1F0), proton leak (H+leak), adenine nucleotide transporter (ANT), Pi-H^+^ transporter (PiHt), K^+^ - H^+^ transport (KHt), adenylate kinase (AK), creatine kinase (CK), lumped cellular ATPase fluxes (ATPase), ubiquinone (Q), ubiquinol (QH2), oxidized cytochrome C (cytC Oxid), reduced cytochrome C (cytC Red), adenosine diphosphate (ADP), inorganic phosphate (Pi), adenosine triphosphate (ATP), nicotinamide adenine dinucleotide (NAD), reduced nicotinamide adenine dinucleotide (NADH), adenosine monophosphate (AMP).

A detailed biophysical model of mitochondrial oxidative ADP phosphorylation previously described by Wu et al. [17] was used as the basis for the present computational study. The model distinguished three cellular compartments: mitochondrial matrix, mitochondrial inter membrane space and cell cytoplasm. A schematic representation of the model is provided in Figure 7.

Model parameterization was updated according to the results of a previous study [14]: dehydrogenase activity (*X_DH*), ANT activity (*X_ANT*) and ANT parameter (*θ*) were set to 0.269 mol s^−1^ M^−1^ (L mito)^−1^, 0.041 mol s^−1^ (L mito)^−1^ and 1, respectively. In addition, proton leak activity (*X_Hle*) was adjusted from 200 mol s^−1^ M^−1^ mV^−1^ (L mito)^−1^ to 33 mol s^−1^ M^−1^ mV^−1^ (L mito)^−1^. The rationale behind this adjustment was to decrease the fraction of protons entering the matrix through the leak in resting skeletal muscle (ATPase = 0.01 mM/s) to values within the range observed experimentally (35–50%) [22]. Adjusting the value of *X_Hle* decreased this fraction from 92% in the original parameterization to 50% according to the new parameterization.

Ordinary differential equations were implemented in Matlab (version 7.5.0; Mathworks, Natick, MA) and numerically solved using ODE15s with relative and absolute tolerance set to 10^−8^ and 10^−8^ respectively. A copy of the model will be made available at the *Biomodels* repository.

### Simulation and parameter estimation protocols

#### Testing of initial model, Figure 1A

The ΔG_p_ – J_p_ relation was calculated from a series of steady state simulations, hereto the ATP consumption in the cytoplasm was incrementally increased (starting value: 0.01 mM/s, step size: 0.01 mM/s) until the steady state cytoplasmic ADP concentration exceeded a level of 0.1 mM. Simulations were run for 10^8^ s to ensure a steady state was reached. Next, ATP consumption was decreased to 0.01 mM/s in order to simulate post exercise recovery conditions.

A list of the adjustable model parameters, their initial and optimized values is provided in the supplementary materials. Parameter *θ* represents a fraction; therefore the value of this parameter was constrained between 0 and 1. Parameter estimation was performed using Matlab routine *lsqnonlin* (Levenberg - Marquardt algorithm) with option *DiffminChange* set to 10^−6^. All other options were set to default values. The error between experimental data and model predictions of steady state behavior as well as post exercise recovery conditions were used to objective function. The parameter estimation procedure was run 100 times, every time randomly perturbing the initial parameter values by +/−10 percent. The model fit with the overall lowest mean squared error was selected as final parameter set.

#### Testing of initial model, Figure 1B

The ΔG_p_ – J_p_ relation was calculated as described in ‘*Testing of initial model,* *Figure 1A* *’.* 10 000 simulations were performed in a Monte Carlo simulation approach randomly selecting parameter values within the range of 0.1–2 times the values of the initial model parameterization. Parameter *θ* represents a fraction; therefore the value of this parameter was constrained between 0 and 1. A list of initial model parameters is provided in the supporting information materials (Table S1, column: value original model). A few parameter sets caused numerical problems when solving the ODE system. These were automatically stopped by the algorithm, excluded from further analyses and substituted by a new simulation. Overall less than 5 percent of the simulations required such substitution. The goodness of fit was quantified by calculating the mean squared error between model simulations and experimental data.

#### Metabolic control analysis, Figure 2

The model parameters included in the metabolic control analysis are listed in Table 1. This list was constructed by selecting all parameters representing enzyme activities (V_max_). Enzyme activity parameters of creatine kinase, adenylate kinase, the mitochondrial K^+^/H^+^ exchanger and magnesium binding fluxes were excluded from the analysis since at steady state the fluxes through these enzymes were zero and consequently, control coefficients could not be calculated. The flux and concentration control coefficients were calculated for an increase in enzyme activity of one percent.

Flux control coefficients were calculated according to Eqn 8 [20]. 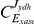, 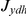 and 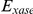, denote the flux control coefficient, a flux through a particular reaction (*ydh*), and an enzyme concentration (*xase*), respectively.
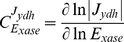
(8)Concentration control coefficients were calculated according to Eqn 9 [20]. 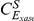, 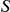 and 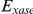, denote the concentration control coefficient, the concentration of a particular metabolite, and an enzyme concentration (*xase*), respectively.
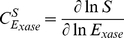
(9)It has been verified that all flux control coefficients summed to 1 and all concentration control coefficients summed to 0.

#### Parameter estimation substrate feedback and parallel activation models

Model parameters were estimated in a two step approach. The first step was selection of initial model parameter values. A wide range of parameter values was explored in a Monte Carlo simulation approach randomly drawing parameter values within selected ranges. The width of the range set for each individual parameter of the substrate feedback model (model conf. *i*) are listed in supporting information Table S4 and parameter ranges explored for the parallel activation model (model conf. *ii*) are listed supporting information Table S5. For each model configurations, in total, 50 000 simulations were run.

The ΔG_p_ – J_p_ relation was calculated as described in ‘*Testing of initial model,* *Figure 1A* *’.* The set of parameters that yielded the best fit to the experimental ΔG_p_ – J_p_ relation, quantified by the mean least square error was then selected as starting point for the second step in the parameter estimation procedure. In this next step, model parameter values were optimized by applying a non linear least square optimization algorithm: i.e., Matlab routine *lsqnonlin* (Levenberg - Marquardt algorithm) with option *DiffminChange* set to 10^−6^. All other options were set to default values. The error between experimental data and model predictions of steady state behavior as well as post exercise recovery conditions were used to objective function. The optimization algorithm was started 100 times, every time adding +/−25% of random noise to the parameter values obtained in step one. The optimal parameter values obtained in this second step are listed in Tables 3 and 4 as the mean and standard deviation of the 100 optimization runs.

#### Model testing, Figure 3

The ΔG_p_ – J_p_ relation was calculated as described in ‘*Testing of initial model,* *Figure 1A* *’.* 1000 simulations were run and parameter values were randomly selected from the 95% confidence interval (mean+/−2*SD) of a normal distributions with mean and SD as reported in Tables 3 and 4. The selected parameters were limited to +/−2×SD, to ensure no negative parameter values were drawn. The solution space of the model was represented by the mean and standard deviation of the 1000 simulations. It was verified that 1000 simulations were enough to obtain a stable solution.

#### Predictions of mitochondrial redox state and membrane potential, Figure 4

The relations between mitochondrial redox potential and ATPase rate and membrane potential and ATPase rate were calculated for steady state conditions. Different steady states were obtained by incrementally increasing the cytoplasmic ATPase rate (starting value: 0.01 mM/s, step size: 0.01 mM/s). Simulations were run for 10^8^ s to ensure a steady state was reached. The simulations were run in a Monte Carlo simulation approach as described in *‘Model testing,* *Figure 3*’.

#### Model testing, Figure 5

The ΔG_p_ – J_p_ relation was calculated for post exercise recovery conditions. The ATP consumption in the cytoplasm was incrementally increased (starting value: 0.01 mM/s, step size: 0.01 mM/s) until the steady state cytoplasmic ADP concentration exceeded a level of 0.065 mM. Simulations were run for 10^8^ s to ensure a steady state was reached. Next, ATP consumption was decreased to 0.01 mM/s in order to simulate post exercise recovery conditions.

The mitochondrial density for simulations of sedentary subjects was set to 0.0235 percent and 0.030 percent for model configuration *i* and *ii*, respectively. The mitochondrial density for simulations of athletes was set to 0.085 percent and 0.080 percent for model configuration *i* and *ii*, respectively.

#### Prediction of ^31^P observed metabolite dynamics during post – exercise recovery period, Figure 6

PCr and Pi recovery are sensitive to cellular pH. Simulations of these metabolite dynamics therefore required to take pH dynamics into account. pH was modeled as described by Kemp et al. [46], [47], Eqn 10.
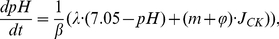
(10)where
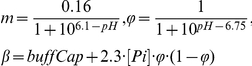
in which *λ* denotes the apparent proton efflux rate parameter, *J_CK_* the flux through creatine kinase and *buffCap* the cytosolic buffer capacity (20 slykes (i.e. mmol·L^−1^·pH^−1^)).

The simulations comprised two parts: i.e., initialization and recovery. The mitochondrial volume percent was set to the values also used to determine the ΔG_p_ – J_p_ in Figure 5. During the initialization of the model the pH was clamped at the experimentally observed end – exercise pH. ATPase demand flux was adjusted to obtain steady state predictions of [PCr] and [Pi] which matched the experimentally observed end – exercise conditions. The steady state values were used as initial conditions of the recovery simulations. During recovery ATPase demand flux was set to resting values (0.01 mM/s). pH dynamics were modeled by Eqn 10. The proton efflux parameter, *λ*, was adjusted to reproduce the experimentally observed pH dynamics. Simulations were performed for the original model and model configuration *i* and *ii* and compared to an individual dataset of a control subject, athlete and subject with sedentary lifestyle. The total creatine concentration was calculated from experimentally observed resting PCr concentration assuming that 15% of the total creatine is unphosphorylated at rest [42]. The parameter settings (ATPase, mitochondrial density, *λ* and total creatine concentration) for all these simulations is listed in the supporting information (Table S6)

## Supporting Information

Figure S1 Effect of variation in end – exercise pH on observed ΔG_p_ – J_p_ relation.
(PDF)

Figure S2 ΔG_p_ – J_p_ and ADP – J_p_ relation according to the initial reparameterized model and model configuration *i* and *ii*.
(PDF)

Figure S3 Predictions of ΔG_p_ – J_p_ and ADP – J_p_ according to the initial model after optimization of model parameters on both the ΔG_p_ – J_p_ and ADP – J_p_ experimental data.
(PDF)

Table S1 Adjustable model parameters.
(PDF)

Table S2 Flux control coefficients.
(PDF)

Table S3 Concentration control coefficients.
(PDF)

Table S4 Range of parameter values explored in Monte Carlo simulation approach (model configuration *i*).
(PDF)

Table S5 Range of parameter values explored in Monte Carlo simulation approach (model configuration *ii*).
(PDF)

Table S6 Parameter settings for the simulations of post exercise recovery dynamics (Figure 6).
(PDF)
