# Supplementary material for: Prediction of Muscle Energy States at Low Metabolic Rates Requires Feedback Control of Mitochondrial Respiratory Chain Activity by Inorganic Phosphate
Source: PLoS One. 2012 Mar 28;7(3):e34118. doi: 10.1371/journal.pone.0034118 (PMC3314597; doi:10.1371/journal.pone.0034118)
Supplement: Figure S1 — Effect of variation in end – exercise pH on observed ΔGp – Jp relation. (PDF) [file pone.0034118.s001.pdf]

Figure S1

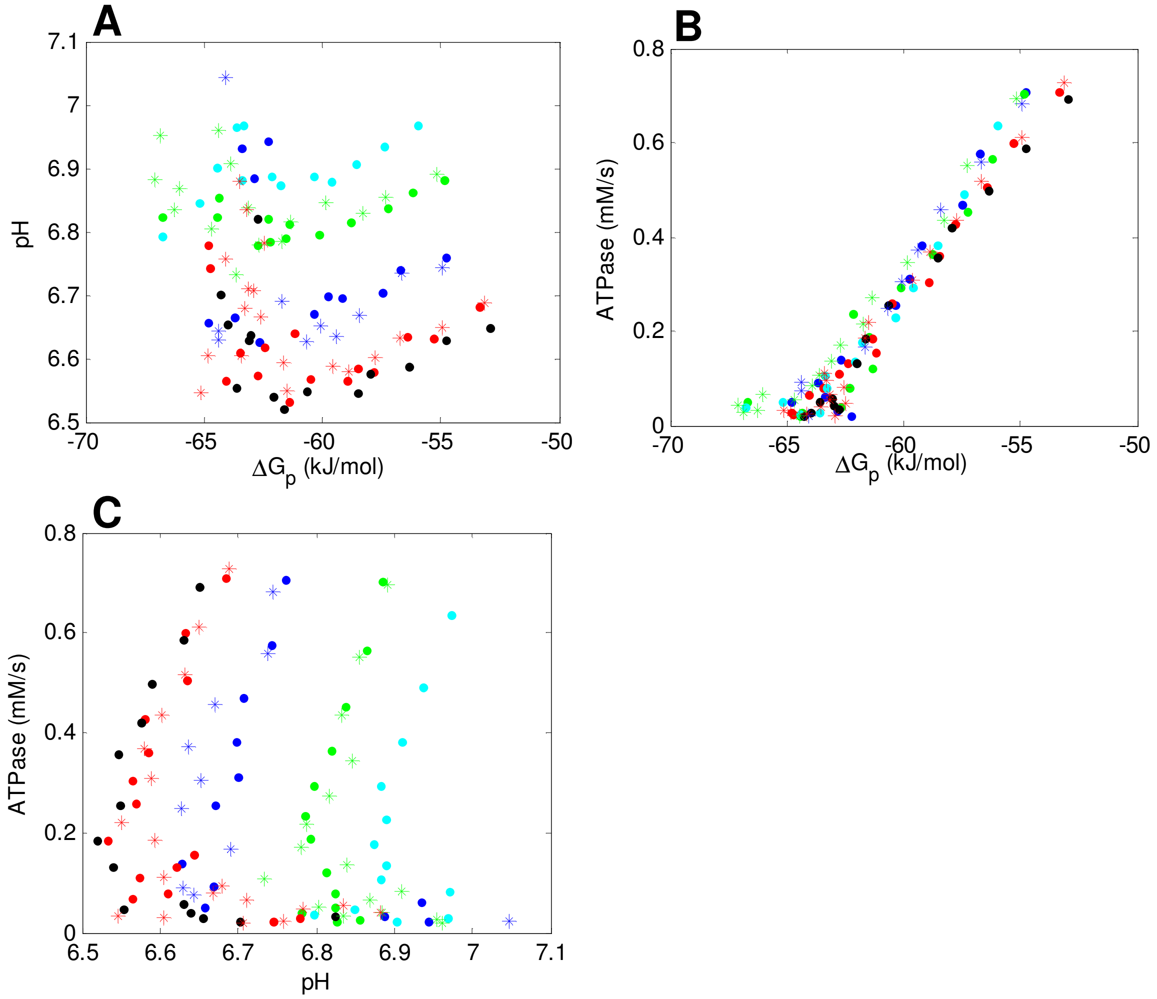

Figure 1, effect of variation in end – exercise pH on observed $\triangle G_p$ – J_p relation. The $\triangle G_p$ - pH (**A**), $\triangle G_p$ – J_p (**B**) and pH – J_p (**C**) of different individual datasets are shown. Each dataset is indicated by specific color – marker combination. These results show that between datasets a large variation in cellular pH values was observed (panel A and C) while the $\triangle G_p$ – J_p relation (B) determined for each dataset was very similar. From these results it was concluded that the $\triangle G_p$ – J_p relation was insensitive to cellular pH.
