# Supplementary material for: Prediction of Muscle Energy States at Low Metabolic Rates Requires Feedback Control of Mitochondrial Respiratory Chain Activity by Inorganic Phosphate
Source: PLoS One. 2012 Mar 28;7(3):e34118. doi: 10.1371/journal.pone.0034118 (PMC3314597; doi:10.1371/journal.pone.0034118)
Supplement: Figure S2 — ΔGp – Jp and ADP – Jp relation according to the initial reparameterized model and model configuration i and ii. (PDF) [file pone.0034118.s002.pdf]

Figure S2

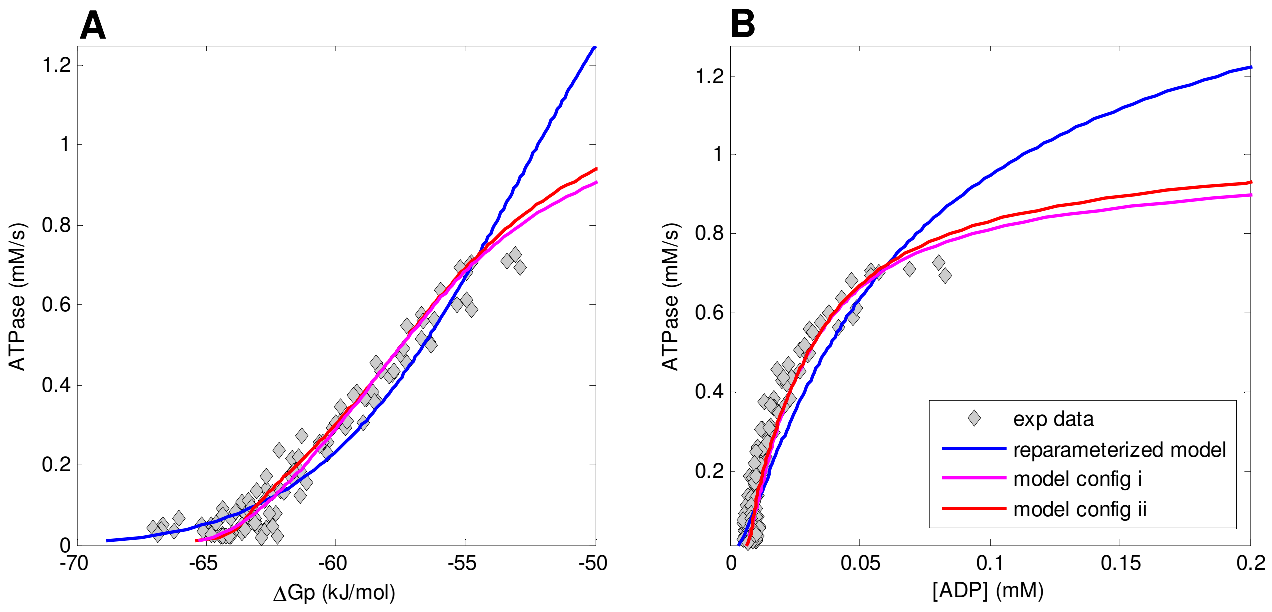

Figure 2, predictions according to the reparameterized model (blue lines), model configuration *i* (magenta lines) and model configuration *ii* (red lines) compared to experimental data (grey diamonds) for the $\triangle G_p - J_p$ relation (**A**) and the ADP – J_p relation (**B**). The simulations of the $\triangle G_p - J_p$ relation show that the reparameterized model did not follow the expected sigmoidal relation. In addition, panel B shows that the reparameterized model failed in reproducing the ADP – J_p relation. In contrast, the $\triangle G_p - J_p$ transduction function predicted according to model configuration *i* and *ii* followed a sigmoidal relation. Furthermore, both model configurations reproduced the ADP – J_p relation with a highly improved accuracy.
