# Supplementary material for: Prediction of Muscle Energy States at Low Metabolic Rates Requires Feedback Control of Mitochondrial Respiratory Chain Activity by Inorganic Phosphate
Source: PLoS One. 2012 Mar 28;7(3):e34118. doi: 10.1371/journal.pone.0034118 (PMC3314597; doi:10.1371/journal.pone.0034118)
Supplement: Figure S3 — Predictions of ΔGp – Jp and ADP – Jp according to the initial model after optimization of model parameters on both the ΔGp – Jp and ADP – Jp experimental data. (PDF) [file pone.0034118.s003.pdf]

Figure S3

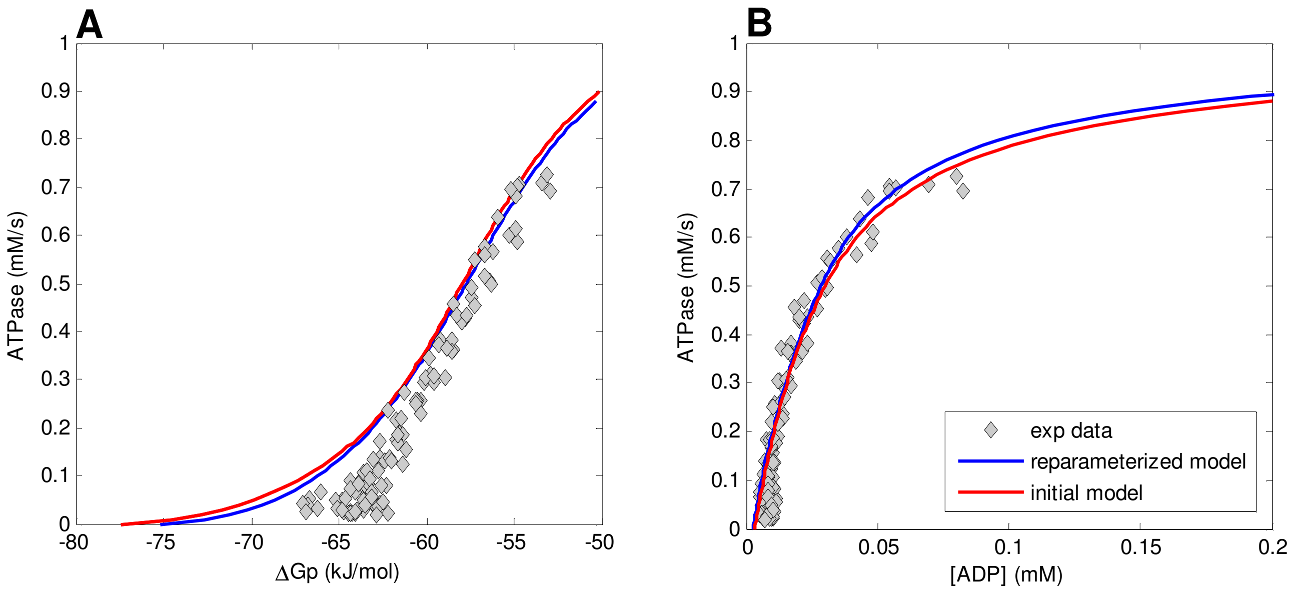

Figure 3, predictions according to the initial model (red lines; the same result as the red line in Figure 1 in the main text) and reparameterized model (blue lines) compared to experimental data (grey diamonds) sampling the $\triangle G_p - J_p$ (**A**) and ADP – J_p relation (**B**). The reparameterized model was obtained by including both the $\triangle G_p - J_p$ and ADP – J_p data in the parameter estimation procedure. The transduction function of the reparameterized model became very similar to the initial model and was rejected because it failed to describe rest and low exercise conditions.
