# Supplementary material for: Prediction of Muscle Energy States at Low Metabolic Rates Requires Feedback Control of Mitochondrial Respiratory Chain Activity by Inorganic Phosphate
Source: PLoS One. 2012 Mar 28;7(3):e34118. doi: 10.1371/journal.pone.0034118 (PMC3314597; doi:10.1371/journal.pone.0034118)
Supplement: Table S1 — Adjustable model parameters. (PDF) [file pone.0034118.s004.pdf]

Table S1, adjustable model parameters,

Parameter name	Description	Value original model	Value optimized model (Figure 1A)	Parameter range sampled in MC simulations (Figure 1B)	Unit
R	Dehydrogenase parameter	4.559	7.61	0.4559 – 9.118	unitless
$k_{Pi,1}$	Dehydrogenase parameter	0.1553	0.317	0.01553 – 0.3106	mM
$k_{Pi,2}$	Dehydrogenase parameter	0.8222	3.70	0.08222 – 1.6444	mM
X_{DH}	Dehydrogenase activity	0.16	0.735	0.016 – 0.32	mol s^{-1} M^{-1} (L mito)$^{-1}$
X_{C1}	Complex I activity	4405	14057	440.5 – 8810	mol s^{-1} M^{-2} (L mito)$^{-1}$
X_{C3}	Complex III activity	4.887	4.10	0.4887 – 9.774	mol s^{-1} $M^{-3/2}$ (L mito)$^{-1}$
$k_{Pi,3}$	Complex III parameter	0.3601	0.560	0.03601 – 0.7202	mM
$k_{pi,4}$	Complex III parameter	5.651	0.334	0.5651 – 11.302	mM
X_{C4}	Complex IV activity	6.766×10^{-5}	10.6×10^{-5}	6.766×10^{-6} – 1.3532×10^{-4}	mol s^{-1} M^{-1} (L mito)$^{-1}$
k_{O2}	Complex IV parameter	0.12	0.11	0.012 – 0.24	mM
X_{F1}	F_oF_1-ATPase activity	1000.0	699.8	100 – 2000	mol s^{-1} M^{-1} (L mito)$^{-1}$
X_{ANT}	ANT activity	0.041	0.112	0.0041 – 0.082	mol s^{-1} (L mito)$^{-1}$
$K_{m,ADP}$	Michaelis constant ANT	3.5×10^{-3}	13.0×10^{-3}	3.5×10^{-4} – 7.0×10^{-3}	mM
k_{PiHt}	H^+/Pi^- cotransporter parameter	0.2542	0.270	0.02542 – 0.5084	mM
X_{PiHt}	H^+/Pi^- cotransporter activity	3.850×10^{-5}	18.9×10^{-5}	3.850×10^{-6} – 7.7×10^{-5}	mol s^{-1} M^{-1} (L mito)$^{-1}$
X_{KH}	K^+/H^+ antiporter activity	5.651×10^{7}	11.9×10^{7}	5.651×10^{6} – 1.1302×10^{8}	mol s^{-1} M^{-2} (L mito)$^{-1}$

P_{pi}	Mitochondrial membrane permeability to inorganic phosphate	327	796.5	32.7 – 654	μm s^{-1}
P_A	Mitochondrial outer membrane permeability to nucleotides	85.0	117.2	8.5 – 170	μm s^{-1}
C_{tot}	total cytochrom C concentration	2.7	3.97	0.27 – 5.4	mmol (L IM water)$^{-1}$
Q_{tot}	total ubiquinol concentration - Q+QH2	1.35	1.26	0.135 – 2.7	mmol (L matrix water)$^{-1}$
NAD_{tot}	total matrix NAD(H) concentration - NAD+NADH	2.97	2.59	0.297 – 5.94	mmol (L matrix water)$^{-1}$
X_{buff}	matrix buffering coefficient	100	118.64	10 – 200	M^{-1}
K_{DT}	Mg-ATP binding constant	24×10^{-6}	231.9×10^{-6}	2.4×10^{-6} - 48×10^{-6}	M
K_{DD}	Mg-ADP binding constant	347×10^{-6}	309.1×10^{-6}	34.7×10^{-6} - 694×10^{-6}	M
gamma	mito membrane area per cell volume	5.99	15.1	0.599 – 11.98	μm^{-1}
θ	ANT parameter	1	0.960	0 – 1	unitless
Total matrix ADP+ATP	Total matrix ADP+ATP	10	8.66	0.1 – 2	mmol (L matrix water)$^{-1}$
