# Supplementary material for: Prediction of Muscle Energy States at Low Metabolic Rates Requires Feedback Control of Mitochondrial Respiratory Chain Activity by Inorganic Phosphate
Source: PLoS One. 2012 Mar 28;7(3):e34118. doi: 10.1371/journal.pone.0034118 (PMC3314597; doi:10.1371/journal.pone.0034118)
Supplement: Table S2 — Flux control coefficients. (PDF) [file pone.0034118.s005.pdf]

<u>Table S2</u><u>, flux control coefficients,</u> ATPase rate 0.01mM/s

	DH flux	C1 flux	C3 flux	C4 flux	F1F0 flux	ANT flux	Pi H+ cotransp flux	Hleak flux	AMP transp flux	ADP transp flux	ATP transp flux	Pi transp flux	ATP cons. Flux
**X_DH**	7.88E-04	7.88E-04	7.88E-04	7.88E-04	3.91E-09	-1.61E-12	3.75E-12	1.66E-03	-5.64E-03	2.82E-06	1.41E-06	-2.98E-11	-6.69E-06
**X_C1**	6.85E-04	6.85E-04	6.85E-04	6.85E-04	1.95E-09	3.03E-12	8.93E-12	1.44E-03	-5.06E-03	2.53E-06	1.27E-06	-5.98E-11	-6.82E-06
**X_C3**	1.38E-02	1.38E-02	1.38E-02	1.38E-02	-9.27E-09	-2.14E-12	-5.36E-13	2.91E-02	-1.02E-01	5.08E-05	2.54E-05	-2.98E-11	2.12E-05
**X_C4**	2.05E-03	2.05E-03	2.05E-03	2.05E-03	3.91E-09	-4.82E-12	-7.14E-13	4.31E-03	-1.51E-02	7.55E-06	3.77E-06	-5.98E-11	-3.94E-06
**X_F1F0**	-1.27E-06	-1.27E-06	-1.27E-06	-1.27E-06	1.05E-08	-4.64E-12	5.53E-12	-2.67E-06	-7.05E-06	3.52E-09	1.99E-09	0.00E+00	-8.32E-06
**X_ANT**	-3.28E-04	-3.28E-04	-3.28E-04	-3.28E-04	1.76E-08	8.93E-12	-1.25E-12	-6.92E-04	-7.35E-03	3.67E-06	1.84E-06	-5.98E-11	-6.19E-06
**X_H+Pi+**	1.16E-05	1.16E-05	1.16E-05	1.16E-05	1.56E-08	3.57E-12	9.64E-12	2.46E-05	-2.08E-03	1.04E-06	5.21E-07	-2.98E-11	-7.72E-06
**X_Hle**	4.67E-01	4.67E-01	4.67E-01	4.67E-01	3.91E-08	4.46E-12	1.39E-11	9.83E-01	6.09E-02	-3.05E-05	-1.52E-05	-5.98E-11	-2.60E-05
**mito Adn**	-7.95E-04	-7.95E-04	-7.95E-04	-7.95E-04	2.93E-08	2.50E-12	6.96E-12	-1.68E-03	4.89E-02	-2.45E-05	-1.22E-05	-2.98E-11	-2.88E-06
**mito Pi**	-8.97E-07	-8.97E-07	-8.97E-07	-8.97E-07	3.27E-08	3.57E-13	1.02E-11	-1.89E-06	-4.79E-05	2.40E-08	1.24E-08	-4.61E-11	-8.31E-06
**X_AtC**	5.19E-01	5.19E-01	5.19E-01	5.19E-01	1.00E+00	1.00E+00	1.00E+00	-1.69E-02	1.02E+00	1.00E+00	1.00E+00	1.00E+00	1.00E+00
**sum**	1.00	1.00	1.00	1.00	1.00	1.00	1.00	1.00	1.01	1.00	1.00	1.00	1.00
