# Supplementary material for: Prediction of Muscle Energy States at Low Metabolic Rates Requires Feedback Control of Mitochondrial Respiratory Chain Activity by Inorganic Phosphate
Source: PLoS One. 2012 Mar 28;7(3):e34118. doi: 10.1371/journal.pone.0034118 (PMC3314597; doi:10.1371/journal.pone.0034118)
Supplement: Table S3 — Concentration control coefficients. (PDF) [file pone.0034118.s006.pdf]

Table S3, concentration control coefficients, ATPase rate 0.01mM/s

	H_x	K_x	Mg_x	NADH	Q	Cox	ATP_x	ADP_x	ATP_mx	ADP_mx	Pi_x	ATP_i	ADP_i	AMP_i
**X_DH**	-1.02E-05	1.64E-04	2.21E-03	1.23E-01	-2.36E-02	-3.45E-04	-8.95E-04	3.75E-03	-7.75E-04	4.76E-03	-4.11E-02	-6.68E-06	-6.10E-03	-1.22E-02
**X_C1**	1.10E-05	-1.78E-04	1.84E-03	-1.47E-03	-2.11E-02	-3.17E-04	-7.49E-04	3.14E-03	-6.48E-04	3.98E-03	-3.65E-02	-6.82E-06	-5.48E-03	-1.09E-02
**X_C3**	2.03E-04	-3.28E-03	3.72E-02	-2.96E-02	3.18E-01	-6.38E-03	-1.51E-02	6.33E-02	-1.31E-02	8.02E-02	-7.35E-01	2.12E-05	-1.10E-01	-2.19E-01
**X_C4**	3.10E-05	-4.99E-04	5.50E-03	-4.38E-03	4.72E-02	2.56E-02	-2.23E-03	9.37E-03	-1.93E-03	1.19E-02	-1.09E-01	-3.94E-06	-1.63E-02	-3.26E-02
**X_F1F0**	9.58E-07	-1.54E-05	9.43E-07	-3.61E-07	-9.29E-08	-2.67E-07	-3.83E-07	1.61E-06	-3.31E-07	2.03E-06	-1.37E-05	-8.32E-06	-1.65E-05	-2.47E-05
**X_ANT**	3.47E-05	-5.59E-04	2.15E-02	-1.49E-03	-5.71E-03	1.17E-04	-8.72E-03	3.66E-02	-7.55E-03	4.63E-02	-4.05E-02	-6.19E-06	-7.95E-03	-1.59E-02
**X_H+Pi+**	-3.64E-07	5.86E-06	-8.38E-04	5.87E-05	2.26E-04	-4.92E-06	3.40E-04	-1.43E-03	2.95E-04	-1.81E-03	1.59E-03	-7.72E-06	-2.26E-03	-4.50E-03
**X_Hle**	-1.09E-04	1.76E-03	-2.22E-02	-4.20E-02	-1.53E-01	-8.86E-03	9.00E-03	-3.77E-02	7.79E-03	-4.78E-02	4.39E-01	-2.60E-05	6.58E-02	1.31E-01
**mito Adn**	8.28E-05	-1.33E-03	5.21E-02	-3.62E-03	-1.39E-02	2.85E-04	-2.12E-02	8.87E-02	-1.83E-02	1.12E-01	-9.83E-02	-4.55E-05	1.39E-01	2.78E-01
**mito Pi**	9.31E-07	-1.50E-05	-1.49E-05	1.39E-06	6.63E-06	-4.09E-07	6.05E-06	-2.54E-05	5.24E-06	-3.22E-05	3.38E-05	-8.31E-06	-6.07E-05	-1.13E-04
**X_AtC**	-2.37E-04	3.81E-03	-9.80E-02	-4.15E-02	-1.50E-01	-1.02E-02	3.97E-02	-1.67E-01	3.44E-02	-2.11E-01	6.24E-01	7.07E-06	-5.72E-02	-1.12E-01
**sum**	0.00	0.00	0.00	0.00	0.00	0.00	0.00	0.00	0.00	0.00	0.00	0.00	0.00	0.00

continued …

	ATP_mi	ADP_mi	Pi_i	Mg_i	ATP_c	ADP_c	AMP_c	ATP_mc	ADP_mc	Pi_c	Mg_c	PCr_c	d_Psi	NADH/NAD
**X_DH**	-6.89E-07	-6.06E-03	-4.03E-02	5.21E-05	-6.69E-06	-5.26E-03	-1.05E-02	-5.49E-06	-5.25E-03	-4.03E-02	5.24E-05	2.59E-04	1.66E-03	1.63E-03
**X_C1**	-7.66E-07	-5.44E-03	-3.63E-02	5.26E-05	-6.82E-06	-4.72E-03	-9.44E-03	-5.62E-06	-4.71E-03	-3.63E-02	5.25E-05	2.32E-04	1.44E-03	1.46E-03
**X_C3**	1.33E-05	-1.10E-01	-7.30E-01	-6.83E-05	2.12E-05	-9.46E-02	-1.89E-01	2.14E-05	-9.46E-02	-7.30E-01	9.21E-06	4.66E-03	2.91E-02	2.95E-02
**X_C4**	6.93E-07	-1.63E-02	-1.08E-01	4.03E-05	-3.94E-06	-1.41E-02	-2.81E-02	-2.84E-06	-1.41E-02	-1.08E-01	4.82E-05	6.92E-04	4.31E-03	4.37E-03
**X_F1F0**	-1.51E-06	2.21E-05	-3.84E-05	5.92E-05	-8.32E-06	-1.43E-05	-2.02E-05	-7.07E-06	-4.10E-07	-3.84E-05	5.50E-05	2.93E-07	-2.68E-06	1.13E-06
**X_ANT**	-4.39E-07	-7.91E-03	-4.09E-02	5.00E-05	-6.19E-06	-6.85E-03	-1.37E-02	-5.01E-06	-6.84E-03	-4.08E-02	5.16E-05	3.37E-04	-6.92E-04	1.70E-03
**X_H+Pi+**	-1.21E-06	-2.22E-03	-1.67E-02	5.66E-05	-7.72E-06	-1.95E-03	-3.89E-03	-6.49E-06	-1.93E-03	-1.67E-02	5.40E-05	9.55E-05	2.46E-05	6.66E-04
**X_Hle**	-1.04E-05	6.58E-02	4.36E-01	1.36E-04	-2.60E-05	5.67E-02	1.13E-01	-2.41E-05	5.67E-02	4.35E-01	8.24E-05	-2.79E-03	-1.74E-02	-1.76E-02
**mito Adn**	-2.03E-05	1.39E-01	-9.91E-02	2.19E-04	-2.88E-06	-1.66E-02	-3.32E-02	-1.85E-06	-1.66E-02	-9.90E-02	4.52E-05	8.17E-04	-1.68E-03	4.13E-03
**mito Pi**	-1.51E-06	-2.21E-05	9.12E-06	5.92E-05	-8.31E-06	-5.24E-05	-9.64E-05	-7.06E-06	-3.85E-05	-3.69E-04	5.49E-05	2.17E-06	-1.90E-06	1.43E-05
**X_AtC**	6.23E-06	-5.72E-02	6.40E-01	-7.31E-06	-3.60E-05	8.86E-02	1.77E-01	-3.37E-05	8.86E-02	6.41E-01	9.78E-05	-4.36E-03	-1.69E-02	-2.61E-02
**sum**	0.00	0.00	0.00	0.00	0.00	0.00	0.00	0.00	0.00	0.00	0.00	0.00	0.00	0.00
