# Supplementary material for: Prediction of Muscle Energy States at Low Metabolic Rates Requires Feedback Control of Mitochondrial Respiratory Chain Activity by Inorganic Phosphate
Source: PLoS One. 2012 Mar 28;7(3):e34118. doi: 10.1371/journal.pone.0034118 (PMC3314597; doi:10.1371/journal.pone.0034118)
Supplement: Table S4 — Range of parameter values explored in Monte Carlo simulation approach (model configuration i). (PDF) [file pone.0034118.s007.pdf]

Table S4, Range of parameter values explored in Monte Carlo simulation approach. This parameter range was explored for the model of regulation by substrate feedback (model configuration *i*).

Parameter name	Parameter range	Unit
X_{CIII}	0 – 100	mol s^{-1} $M^{-3/2}$ (L mito)$^{-1}$
nH	0 – 10	Unitless
K_{50Pi}	0 – 30	mM
