# Supplementary material for: Prediction of Muscle Energy States at Low Metabolic Rates Requires Feedback Control of Mitochondrial Respiratory Chain Activity by Inorganic Phosphate
Source: PLoS One. 2012 Mar 28;7(3):e34118. doi: 10.1371/journal.pone.0034118 (PMC3314597; doi:10.1371/journal.pone.0034118)
Supplement: Table S5 — Range of parameter values explored in Monte Carlo simulation approach (model configuration ii). (PDF) [file pone.0034118.s008.pdf]

Table S5, Range of parameter values explored in Monte Carlo simulation approach. This parameter range was explored for the model of regulation by parallel activation (model configuration *ii*).

Parameter name	Parameter range	Unit
X_A	0 -10	Unitless
X_{IA}	0 – 1	Unitless
nH	1 – 4	Unitless
K_{50AtC}	0 – 0.1	mmol (L cell water)$^{-1}$ s^{-1}
K_{on}	0 – 1	s^{-1}
K'_{off}	0 – 1000	s^{-1}
