# Supplementary material for: Prediction of Muscle Energy States at Low Metabolic Rates Requires Feedback Control of Mitochondrial Respiratory Chain Activity by Inorganic Phosphate
Source: PLoS One. 2012 Mar 28;7(3):e34118. doi: 10.1371/journal.pone.0034118 (PMC3314597; doi:10.1371/journal.pone.0034118)
Supplement: Table S6 — Parameter settings for the simulations of post exercise recovery dynamics (Figure 6). (PDF) [file pone.0034118.s009.pdf]

Table S6, parameter settings for the simulations of post exercise recovery dynamics (Figure 6)

Control dataset

Model configuration	Parameter name					
	ATPase demand flux (mmol (L cell water)$^{-1}$)	Mitochondrial volume (mL mito / mL cell)	End exercise pH (unitless)	Cr_{tot} (mmol (L cell water)$^{-1}$)	Pi initial (mmol (L cell water)$^{-1}$))	λ (mM s^{-1} pH $unit^{-1}$)
Original model	0.90	0.056	6.65	41.2	4.0	0.33
Model configuration 1 (substrate feedback)	0.90	0.056	6.65	41.2	4.0	0.33
Model configuration 2 (parallel activation)	0.90	0.056	6.65	41.2	4.0	0.12

Athlete dataset

Model configuration	Parameter name					
	ATPase demand flux (mmol (L cell water)$^{-1}$)	Mitochondrial volume (mL mito / mL cell)	End exercise pH (unitless)	Cr_{tot} (mmol (L cell water)$^{-1}$)	Pi initial (mmol (L cell water)$^{-1}$))	λ (mM s^{-1} pH $unit^{-1}$)
Original model	1.1	0.085	6.95	38.2	4.0	1.33
Model configuration 1 (substrate feedback)	1.1	0.085	6.95	38.2	4.0	1.33
Model configuration 2 (parallel activation)	1.1	0.080	6.95	38.2	4.0	1.33

Subject with sedentary lifestyle dataset

Model configuration	Parameter name

	ATPase demand flux (mmol (L cell water)$^{-1}$)	Mitochondrial volume (mL mito / mL cell)	End exercise pH (unitless)	Cr_{tot} (mmol (L cell water)$^{-1}$)	Pi initial (mmol (L cell water)$^{-1}$))	λ (mM s^{-1} pH unit^{-1})
Original model	0.35	0.0235	6.70	47.0	4.0	0.1
Model configuration 1 (substrate feedback)	0.35	0.0235	6.70	47.0	4.0	0.1
Model configuration 2 (parallel activation)	0.42	0.030	6.70	47.0	4.0	0.017
